# Adverse Effects of Bisphenol A on the Liver and Its Underlying Mechanisms: Evidence from *In Vivo* and *In Vitro* Studies

**DOI:** 10.1155/2022/8227314

**Published:** 2022-08-16

**Authors:** Al-Salihi Ahmed Rashid Abdulhameed, Vuanghao Lim, Hasnah Bahari, Boon Yin Khoo, Muhammad Nazrul Hakim Abdullah, Jun Jie Tan, Yoke Keong Yong

**Affiliations:** ^1^Department of Human Anatomy, Faculty of Medicine and Health Sciences, Universiti Putra Malaysia, 43400 Serdang, Selangor, Malaysia; ^2^Advanced Medical and Dental Institute, Universiti Sains Malaysia, Bertam, 13200 Kepala Batas, Penang, Malaysia; ^3^Institute for Research in Molecular Medicine, Universiti Sains Malaysia, 11800 USM, Penang, Malaysia; ^4^Department of Biomedical Sciences, Faculty of Medicine and Health Sciences, Universiti Putra Malaysia, 43400 Serdang, Selangor, Malaysia

## Abstract

BPA is a known endocrine-disrupting agent that is capable of binding to the estrogen receptor and has exhibited adverse effects in many laboratory animal and in vitro studies. Moreover, it also been shown to have estrogenic, antiandrogenic, inflammatory, and oxidative properties. The widespread presence of BPA in the environment presents a considerable threat to humans. BPA has been shown to be leached into the human ecosystem, where it is commonly found in food products consumed by humans. Although the concentration is relatively low, its prolonged consumption may cause a variety of deleterious health effects. The liver is an important organ for metabolizing and detoxifying toxic metabolites to protect organisms from potentially toxic chemical insults. BPA that is ingested will be eliminated by the liver. However, it has also induced hepatoxicity and injury via various mechanisms. To find research demonstrating the effects of BPA on kidney, a number of databases, including Google Scholar, MEDLINE, PubMed, and the Directory of Open Access Journals, were searched. Thus, this review summarizes the research on the relationship between BPA and its effects on the liver-derived from animals and cellular studies. The underlying mechanism of liver injury caused by BPA is also elucidated.

## 1. Introduction

Bisphenol A (BPA), also known as 2,2-bis(four-hydroxyphenyl) propane, is a popular artificial organic molecule that is utilised in polycarbonate plastics and epoxy resins as an intermediate sort of combination product [1]. BPA is also known as an endocrine disruptor for its ability to mimic the repercussions of endogenous hormones [2]. It represented a global market share of 15 billion pounds in 2013, and it is one of the largest manufacturing chemicals within the global synthetics industry in volume [3]. BPA is used for manufacturing epoxy resins, which are found in plastic food containers, piping, wire insulations, and healthcare consumables. Due to the increased popularity of the production and usage of this product, BPA has been infiltrated and released into our ecosystem and food chain. Therefore, organisms, including humans, are constantly exposed to BPA. A plethora of recent research has shown that a significant population has measurable amounts of BPA in either urine or serum samples, but the importance of existing levels of sensitivity to the toxicological consequences of BPA remains fiercely contested [4–11].

The prime cause of BPA contamination is from the leaching out of plastic products. The sources of BPA ingestion may vary according to environmental, social, and age factors, including baby and beverage bottles, as well as the repeated use of containers, food cans, and even medical equipment such as polycarbonate hemodialysis equipment [12, 13]. Ingestion of these contaminated foods and liquids may bring harm to human health, especially the liver. The liver is critical in the control of several physiological processes of the body, and it is the primary organ engaged in the detoxification of a variety of medications and xenobiotics. Due to that, it plays an important role in the elimination of ingested BPA. It was believed that when unconjugated BPA (the active form of BPA) is taken orally, it is promptly conjugated in the liver and subsequently eliminated via bile or urine [14]. However, the *β*-glucuronidase enzyme, which is present within many tissues, is able to deconjugate BPA [14], and this might result in the bioaccumulation of BPA in the body. In fact, a recent study reported that most plasma BPA is bound to serum protein and the accumulation of BPA was about three times higher in fat compared to other tissues [15]. Numerous evidences from preclinical studies have also proven that the administration of BPA in animals was correlated with the alteration of blood lipid profile and interfered with the oxidant/antioxidant mechanism in the liver [16, 17], which may eventually lead to liver damage.

Since BPA might influence several biological processes associated with liver function, which may lead to liver damage, this review is aimed at providing a concise summary of the data regarding the detrimental effects of BPA exposure on the liver. The analysis of numerous references was considered, many of which included cellular and animal experiments and offered a detailed summary on the topic at hand.

## 2. Methodology for Data Collection

In online research databases like PubMed, Web of Science, MEDLINE, Google Scholar, and Science Direct, articles on the effect of BPA on kidney were searched for using specific keywords such as “bisphenol a,” “BPA,” “kidney,” “in vitro,” “in vivo,” and “animal model.” These articles were then downloaded.

## 3. Experimental Evidence of BPA Damage to the Liver, In Vivo Model

### 3.1. BPA Changed the Level of Liver Enzyme

The liver is the largest internal organ, and it performs many functions, including metabolizing and detoxifying waste products, synthesizing proteins, and maintaining a stable blood glucose level. In order to carry out the abovementioned activities, liver enzymes play the most important role here to speed up these chemical reactions. However, changes in the level of liver enzymes can suggest liver injury or the alteration of bile supply [18]. For instance, aspartate aminotransferase (AST) and alanine aminotransferase (ALT) are the most sensitive indicators of hepatocyte injury [19]. These enzymes will leak into the bloodstream when the hepatocytes are injured or when changes occur in the cell membrane permeability [19]. BPA is known as an endocrine-disrupting agent, and it is also often considered toxic to the body, inducing oxidative stress by impacting vital organs. On top of that, some studies have shown that increased reactive oxygen species (ROS) levels contribute to the oxidation of unsaturated fatty acid cellular constituents and otherwise cause cell apoptosis and degeneration [20].

Some studies investigated the effect of BPA on the liver enzyme in vivo (Table 1). The range of dosages and the duration of exposure time used in the studies vary, from the lowest of 1.0 *μ*g/kg, up to the highest of 120 mg/kg, and the shortest of 1 hour, to the longest of 140 days, respectively. The mode of administration of BPA is mainly via oral but could also be based on intraperitoneal and intravenous injections. Based on the data obtained, BPA significantly affects liver enzyme activity, where it might be able to prime the liver damage. According to Moon and his team [21], a single injection of 1.2 mg/kg b.w./day of BPA significantly elevated AST and ALT levels within 24 hr compared to control in specific pathogen-free C57BL/6 male mice. Hassan et al. reported that 10 and 50 mg/kg of BPA significantly increased serum ALT, ALP, and bilirubin compared to control when albino rats received BPA once daily for up to 4 weeks [22]. On top of that, another two studies also documented that AST and ALT levels significantly increased in BPA-treated rats which received a daily dose of 10 and 50 mg/kg for a duration of 8 weeks compared to control [23, 24]. Moreover, rats treated with 10 mg/kg of BPA for a duration of 60 days also showed significantly increased AST, ALT, and PAL levels when compared to the control group [25]. Interestingly, some studies reported that BPA does not significantly affect liver enzyme activities. For instance, rats treated with 50 and 500 *μ*g/kg/day of BPA for 20 weeks did not induce alteration of AST and ALT levels compared to control [26]. Furthermore, Mourad and Khadrawy [27] also proved that 10 mg/kg/day of BPA showed no significant difference compared to control in AST and ALT activities in 6- or 10-week duration of treatment. However, 25 mg/kg/day (6 weeks of treatment) of BPA showed a significant elevation of AST and ALT activities compared to control. BPA at 4 mg/kg did not show any effect in rat models of hepatic I/R injury after 24 hours postreperfusion [28]. The differences in the results obtained by different researchers could be due to differences in terms of animal strains/species, gender of the animal, dosage, and treatment duration, as well as environmental factors.

### 3.2. Consumption of BPA Altered the Histomorphology of Liver Cells

The harmful effects of BPA on the liver have all been well documented. Studies on several animal models have clearly shown that exposure to BPA, whether short- or long-term, can cause direct hepatotoxicity in the aspect of histomorphology (Table 2). According to Zaulet and his team [34], CD-1 mice treated with 200 mg/kg b.w. daily of BPA orally for only 10 days significantly induced necrotic changes in hepatocytes. The changes were particularly pronounced in the centrilobular area. Inflammatory cell infiltration and vascular congestion were observed in the BPA-treated group compared to the control. Similar results were observed in another study. However, the concentration of BPA was reduced to 130 mg/kg b.w./day for four weeks [35]. On the other hand, studies from the Peerapanyasut Laboratory using 5 and 50 mg/kg b.w./day of BPA in rats for five weeks provided some evidence for a causative role for BPA in liver toxicity, particularly in rats receiving BPA 50 mg/kg b.w./day [29]. Furthermore, Peerapanyasut and his team [31] further investigated the harmful effects of BPA on acute kidney injury-induced remote organ injury in the liver. Based on the data obtained, BPA significantly altered the liver cell structure where dilated sinusoids, centrilobular congestion, leukocyte infiltration in portal tracts, and focal hepatocellular necrosis were observed [31]. Additionally, significant pathological changes in liver cells were also reported in several other studies [25, 30, 36, 37]. More recently, Long and his team [38] documented that gestational exposure to BPA leads to a sex-dependent effect on hepatic lipid accumulation in adult male offspring, and the formation of intracellular lipid corpuscles in the BPA-exposed liver tissue, as revealed by electron microscopy. This evidence suggests deleterious effects on liver function after long-term BPA consumption.

## 4. Mechanism Study of BPA on Liver

Previous studies have unravelled the possible mechanisms underlying the detrimental effects of BPA, particularly on the liver, including evidence derived from in vivo and in vitro experiments, among which are further elaborated hereafter.

### 4.1. Injury from Oxidative Stress

Reactive oxygen species (ROS) are known to have an important role in the physiological functions of the human body. It serves as a second messenger in various cell signalling pathways that control the homeostasis of normal cellular functions [39] and also acts as a promoter of natural defences by killing bacteria [40]. However, when the production of ROS exceeds the capacity of antioxidant defences, oxidative stress is inflicted and can negatively affect the biological tissue function and structural integrity. These events will eventually result in defects and diseases. There are several ways how ROS causes cellular damage, such as damaging DNA through strand breaks and base oxidation, which leads to cell death. The oxidation of proteins results in the malfunctioning of the enzymatic process; the peroxidation of lipids may further propagate maladaptive inflammation by producing proinflammatory mediators, as well as participating in the signalling pathway, which leads to cellular damage [41].

Increasing evidence on BPA exposure suggests liver damage by oxidative stress [22]. Regardless of the presence of the antioxidant system within the liver, BPA causes a significant hepatocellular injury [22]. The study has also shown that BPA at the dose of 50 mg/kg/once daily, orally for four weeks significantly induced liver damage by affecting the oxidant/antioxidant balance, where it increased oxidants, TBARS and NO(x) levels, and decreased antioxidants, CAT, GSH, and SOD levels [22] (Table 3). This is in line with the findings by Kourouma and his team [42], who also demonstrated that BPA induced the formation of ROS and lipid peroxidation, which eventually resulted in cell injury or death. Moreover, ROS that was produced causes oxidative damage to DNA, which can be observed through the TUNNEL assay [42]. If the damaged DNA is not restored successfully and in a timely manner, it could result in teratogenesis, carcinogenesis, mutagenesis, and other irreversible effects [43]. In view of the potential effect of BPA in interfering with redox signalling, an imbalance between oxidants and antioxidants in the liver is inevitable in the presence of BPA.

### 4.2. Mitochondria Dysfunction

Mitochondria is an essential intracellular organelle that serves as a powerhouse of a cell which generates ATP through oxidative phosphorylation (OXPHOS). It also plays a crucial role in controlling of cell death through the activation of the intracellular signalling cascades or death receptor-mediated pathways [44]. Mitochondria is essential in hepatic metabolism, particularly in maintaining the hepatic energy metabolism, an important hub that serves as a critical site for the production and exchange of metabolic intermediates to maintain well-regulated tissue homeostasis in the human body [44].

Mitochondria is among the important targets of endocrine-disrupting agents, including BPA [33, 45–47]. Previous studies have shown that BPA causes a decrease in ATP synthesis in the mitochondria in insulinoma cells in rats [9]. Khan and his team [47] showed that all concentrations (150, 250, and 500 mg/kg b.w./daily, via oral for 14 days) of BPA affected enzyme activities of hepatocyte mitochondria electron transport chain, by significantly decreasing complexes I, II, II, IV, and V in the electron transport chain reaction [47] (Table 3). Another study also reported that activities of the mitochondria complexes I-IV in the liver of the BPA-treated mice were significantly reduced [48]. Moreover, both mitochondrial respiratory chain complex V production and intercellular ATP content were substantially decreased in the liver tissues following BPA ingestion. Data even showed that BPA enhanced the expression of mitochondria apoptotic pathway genes, including caspase-3, caspase-8, caspase-9, and caspase-10, as well as the function of caspase enzymes [48]. A similar study by Moon's laboratory also reported a similar pattern of results [21], albeit the dosages used were much lower than others, which were 0.05 and 1.2 mg/kg b.w/day (below the no observed adverse effect level, NOAEL), administrated intraperitoneally for five days in mice [21]. Both dosages significantly impaired the structure of the hepatic mitochondria, thus leading to hepatocellular injury, despite the low dosage. Furthermore, 10 or 100 nM of BPA also significantly decreased the oxygen consumption rate, ATP production, and mitochondrial membrane potential in HepG2 cells [21] . Xia et al. also indicated that BPA induced mitochondria-mediated apoptosis in hepatic cells, and BPA acts directly on the mitochondria by altering its ultrastructure, inducing permeability transition and releasing proteins that lead to the activation of apoptosis [33]. Collectively, dietary BPA consumption decreased energy generation in hepatocytes by inhibiting mitochondrial respiratory chain complex function and inducing apoptosis via the mitochondrial pathway.

### 4.3. Endoplasmic Reticulum Stress

The endoplasmic reticulum functions to synthesize and fold secretory and membrane proteins. It also serves as a special oxidizing compartment that facilitates the folding of membrane and secretory proteins to be transferred to the cell surface, as well as to the lysosomes and Golgi apparatus. These folded secretory and membrane proteins function to regulate body homeostasis, including acting as hormones, enzymes, signalling molecules, calcium ion buffering, and the biosynthesis of phospholipids and cholesterol [49]. Therefore, the effective and efficient functioning of the endoplasmic reticulum is required for most cellular activities and survival. Recent evidence has shown that there is a link between endoplasmic reticulum with apoptosis, which is associated with the accumulation of misfolded or unfolded proteins (endoplasmic reticulum stress) [50–52]. The number of factors that interrupt endoplasmic reticulum function or disturbances in homeostasis processes, which eventually lead to a state in which protein is misfolded, is depletion of endoplasmic reticulum calcium, changes in the redox status, and energy deprivation [53]. Gene defect increased protein traffic through the endoplasmic reticulum environment, and altered posttranslational alteration is all factors that lead to the aggregation of unfolded protein [53]. These events in the endoplasmic reticulum are linked to a variety of pathophysiological disorders, including cell death [54]. Furthermore, an increasing number of endoplasmic reticulum proteins have been shown to affect apoptosis or cell death by interfering with BCL-2 family members or modifying endoplasmic reticulum calcium ion signalling, whereas some endoplasmic reticulum proteins are caspase substrates that may control the apoptosis execution process [55]. Moreover, recent research on the relationship between stress and apoptosis in the endoplasmic reticulum has shown that it will initiate pathways leading to caspase activation and apoptosis directly [55]. Asahi and his team [2] successfully proved that mouse nonparenchymal hepatocytes treated with 100 *μ*M BPA significantly induced endoplasmic reticulum stress-associated apoptosis in vitro (Table 3). This effect is strongly correlated with endoplasmic reticulum stress biomarkers, such as elevation in the expression of CHOP mRNA, caspase-12, and GRP78/BiP [2]. A morphological examination has also shown significant elongation of the rough endoplasmic reticulum, corroborating the endoplasmic reticulum stress diagnosis [2]. More recently, Figueiredo et al. first showed that BPA exposure in mice causes endoplasmic reticulum stress in the liver in vivo [56]. Taken together, BPA-induced hepatocellular injury or apoptosis is associated with endoplasmic reticulum stress.

### 4.4. Inflammatory Injury

BPA has also been found to be involved in triggering an inflammatory reaction by stimulating the overexpression of a variety of inflammatory-related transcription factor genes and inflammatory-related cytokine genes. Nitric oxide (NO) is a signalling molecule that plays an important role in a wide range of physiological processes, including inflammatory responses. Under normal physiological circumstances, it has an anti-inflammatory influence [57, 58]. NO, on the other hand, is a proinflammatory mediator that, in abnormal conditions, causes inflammation due to excessive production [59, 60]. Regarding the role of NO, there is no definitive reason for NO's position in pro- or antipathophysiologic situations. However, the amount of NO development in the microenvironment may be a deciding factor [61]. According to Byun et al., NO production is altered by the treatment of BPA in vitro and in vivo [62]. They found that, regardless of the dosage, the amount of NO released by peritoneal macrophages from BPA-exposed mice was reduced, but only the 500 mg/kg b.w./day group showed a significant reduction [62]. Byun and his team suggested that BPA exposure in humans, whether in the atmosphere or occupational settings, can increase susceptibility to microbial infections or carcinogenesis due to BPA-induced modulation of NO production [62]. Furthermore, they also demonstrated that the level of TNF-*α* was significantly suppressed with 10 and 100 *μ*M BPA, compared to that of control mice [62]. TNF-*α* is a pleiotropic and potent proinflammatory cytokine that is involved in inflammation, cell growth, differentiation, and apoptosis in the immune system [63]. The reduction of the TNF-*α* production could be associated with the downregulation of NO production. Although TNF-*α* involves mainly inflammation, it is still needed for effective pathogen defence, inflammation resolution, and tissue repair [64]. The homeostatic effect of TNF-*α* and BPA-induced suppression of TNF-*α* production could lead to other pathological conditions.

Male rats administrated with BPA at 50 mg/kg b.w./day, orally for eight weeks, significantly increased in proinflammatory cytokines (IL-1*β*) and at the same time reduced the production of anti-inflammatory/antifibrotic cytokine (IL-10) [23] (Table 3). IL-1ß is secreted from hepatic macrophages in various murine models of liver inflammation [65]. The release of IL-1*β* will further activate macrophages, stimulate the production of other cytokines, and recruit inflammatory cells during infection, injury, and inflammation [66]. Numerous studies have shown that an elevated level of IL-1*β* is closely associated with liver injury, and its suppression might result in hepatoprotective effects during inflammation [67, 68]. On the other hand, IL-10 is an anti-inflammatory cytokine and critical immunoregulatory molecule that keeps the immune system in check, enabling inflammation to be cleared, thus causing the least amount of harm to the host. It is reported that IL-10 is upregulated during macrophage activation in liver injury and thus has a therapeutic role in the downregulation of inflammation in vivo [23]. A study by Moon and his team also demonstrated that BPA-induced liver injuries were associated with the production of inflammatory cytokines in the liver [21]. They measured the hepatic expression of IL-6 and TNF-*α* at 1, 6, and 24 hr after the injection of 1.2 mg/kg/b.w./day of BPA in animals. The results show that the levels of IL-6 and TNF-*α* were increased in the BPA-treated group compared to the control. Wang's laboratory also demonstrated similar results as in Moon's laboratory, where the serum levels of IL-1*β*, IL-6, IL-8, and TNF-*α* in the BPA group were markedly higher than those in the control group [48]. Collectively, BPA is capable of inducing a liver inflammatory response by upregulating pro-inflammatory cytokines, while at the same time downregulating anti-inflammatory cytokines, which in turn leads to liver injury and damage.

## 5. Conclusion and Future Perspective

**Figure 1:**
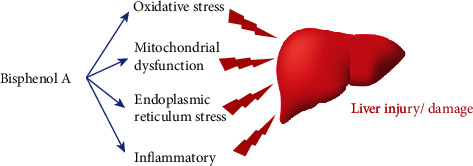
Schematic diagram proposing the possible mechanisms that cause liver injury/damage by BPA.

Extensive scientific research has documented the side effects of BPA on human health, which include affecting neuronal development, reproductive system, endocrine system, metabolism, cardiovascular system, immunological diseases, and even cancer. However, according to the European Food Safety Authority (EFSA), many factors render it difficult to conclude that BPA is harmful to human health, including toxicokinetic variations between animal and human models, various checked routes of exposure, and the nonreproducibility of human experiments on larger scales [69]. Despite these variables, a recent meta-analysis reported a relationship between early BPA exposure and hyperactivity in children. Thus, understanding how BPA works and causes alterations in organs, especially liver injury or damage, can aid in the prevention and diagnosis of the resulting health problems (Figure 1), as well as the development of more effective approaches and technologies for treating BPA-related liver pathological conditions. It is essential to further analyse liver pathologies in rodents and higher mammals with early life BPA exposure at relevant doses and to evaluate whether these pathological changes could trigger or promote cancer formation when coupled with additional carcinogenic insults. Furthermore, it is strongly recommended that the regulation of BPA usage should be revisited to protect human health.

## Figures and Tables

**Table 1:** Summary of selected studies on the effect of BPA on liver enzyme activity. Abbreviations: b.w.: body weight; PEG: polyethylene glycol; IP: intraperitoneal; IV: intravenous; GD: gestation day; NA: not applicable.

No.	Type of animal	Dose/concentration	Vehicles	Mode of administration	Duration of treatment	Liver function test results	Reference
1	Swiss rats	10 mg/kg b.w./day	Water	Oral	60 days/daily/once	Increase in AST, ALT, PAL, LDH, and TNF-alpha	[25]
2	Male Wistar rats	5 and 50 mg/kg b.w./day	Corn oil	Oral	5 weeks/daily/once	5 mg/kg b.w./day—not significant compared to control in both AST and ALT; 50 mg/kg b.w./day—significant compared to control in both AST and ALT	[29]
3	Male Sprague-Dawley rats	4 mg/kg b.w./day	Dissolve in DMSO, diluted with saline	IV	24 hours/once	No significant difference in BPA group compared to control after 24 hr postreperfusion in rat model of hepatic I/R injury	[28]
4	Male C57BL/6 mice	1.0, 10, 50, and 250 *μ*g/kg b.w./day	PEG	Oral	35 days/daily/once	No significant difference compared to control at all concentration for PDH, IL-1 beta, PLA2, AST, and ALT; however, there was significant difference for LDH, ACS, and CPT1 at concentration of 25 *μ*g/kg b.w./day	[30]
5	Male Wistar rats	5 and 50 mg/kg b.w./day	Corn oil	Oral	5 weeks/daily/once	Significant elevation of AST and ALT in the renal ischemia and reperfusion animals when exposed to BPA at concentration of 5 and 50 mg/kg b.w./day	[31]
6	Male Wistar rats	50 and 500 *μ*g/kg b.w./day	Water	Oral	20 weeks	No significant difference compared to control in both concentration for AST and ALT	[26]
7	Male Wistar albino rats	25 and 50 mg/kg b.w./day	NA	Oral	25 mg/kg (6 weeks); 10 mg/kg (6 weeks and 10 weeks); 5 days a week	10 mg/kg b.w./day (6 and 10 weeks) showed no significant difference compared to control in AST and ALT; 25 mg/kg b.w./day (6 weeks) showed significant elevation compared to control	[27]
8	Male Wistar albino rats	0.1, 1, 10, and 50 mg/kg b.w./day	Ethanol in water	Oral	4 weeks/daily/once	10 and 50 mg/kg b.w./day of BPA showed significantly increased in serum ALT, ALP, and bilirubin	[22]
9	Male Wistar rats	5, 25, and 125 mg/kg b.w./day	Olive oil	Oral	35 days/daily/once	All BPA dosages reduced ALP and AST serum levels except ALT	[32]
10	Male Wistar rats	10 mg BPA/kg b.w./day	Olive oil	Oral	8 weeks/daily/once	AST, ALT, ALP, and bilirubin were significantly increased compared to control	[24]
11	Wistar albino rats	50 mg/kg b.w./day	Corn oil	Oral	8 weeks/daily/once	AST and ALT significantly increased in BPA group compared to control	[23]
12	Specific pathogen-free C57BL/6 male mice	1.2 mg/kg b.w./day	Normal saline	IP	1, 6, and 24 hr	No difference after 5 days of treatment (ALT, 60 ± 14 IU/L vs. 57 ± 10 IU/L, *P* > 0.05), although serum AST levels showed an increasing tendency in BPA-treated mice (30 ± 4 U/L vs. 46 ± 20 U/L, *P* > 0.05)	[21]
13	Specific pathogen-free C57BL/6 male mice	1.2 mg/kg b.w./day	Normal saline	IP	1, 6, and 24 hr	Within 24 hr of a single injection of 1.2 mg/kg b.w./day of BPA, both AST and ALT levels significantly increased (*P* = 0.03 and 0.01, respectively)	[21]
14	Virgin female (270–300 g) and male (350–400 g) genitor Wistar rats were	50 *μ*g/kg/b.w./day	Corn oil	Oral	GD 0 to the end of lactation at postnatal day 21	Perinatal exposure to BPA only resulted in a minor increase in serum ALT levels at 3 and 15 weeks but a significant increase at 21 weeks compared to the control in offspring	[33]

**Table 2:** Summary of selected studies on the effect of BPA-induced liver histomorphological changes. Abbreviation: b.w.: body weight; PEG: polyethylene glycol; IV: intravenous.

No.	Type of animal	Dose/concentration	Vehicles	Mode of administration	Duration of treatment	Histology result	Reference
1	Swiss rats	10 mg/kg b.w./day	Water	Oral	60 days/daily/once	BPA group showed steatosis and inflammation. H&E, and oil red O-stained sections showed early indications of hepatic inflammation with Kupffer cell infiltration and steatosis (lipid droplet buildup in hepatocyte cytoplasm).	[25]
2	Sprague-Dawley male rats	25 mg/kg b.w./daily	Sesame oil	Oral	60 days/daily/once	Sinusoidal spaces grew larger, cells lost their typical polygonal form, acidophilus reduced, and hepatocyte cord structure deteriorated. The hepatocyte cytoplasm had a significant number of foamy-like vacuolar degeneration and a big number of apoptotic cells. Oil droplets produced in hepatocytes and the perisunisoidal region were concentrated. The centralis and portal area fibre density and sinusoidal space dilatations were enhanced. The reticular fibre distribution indicated liver fibrosis.	[36]
3	Male Wistar rats	5 and 50 mg/kg b.w./day	Corn oil	Oral	5 weeks/daily/once	BPA 50 mg/kg showed a decrease in mitochondrial number with asymmetric mitochondrial swelling.	[29]
4	Prepubertal female Sprague-Dawley	10 and 100 mg/kg b.w./day	Palm oil	Oral	6 weeks/daily/once	LD BPA group revealed abnormally enlarged sinusoidal cavity, KC, necrotic hepatocytes, granularly degraded hepatocytes, and dilated sinusoids. Massive hepatocytes with cytoplasmic vacuolation or granulation and prominent Kupffer cells were seen in the BPA 100 mg/kg group.	[37]
5	Male CD-1 mice	200 mg/kg b.w./day	Corn oil	Oral	10 days/daily/once	Necrotic hepatocytes in the centrilobular region. Moreover, evident was inflammation and vascular congestion.	[34]
6	Adult C57BL/6J mice	1, 10, 100, and 1000 *μ*g kg b.w./day	Corn oil	Oral	BPA administration was initiated at E7.5, before the development of the embryonic liver and resumed up until E16.5	BPA exposure during pregnancy influenced hepatic fat buildup in adult male offspring. Male offspring treated with BPA gained weight and had higher hepatic TG levels when subjected to HFD. BPA enhanced the amount of HFD-induced hepatic lipid droplets. In BPA-exposed liver tissue, electron imaging indicated an increase in intracellular lipid corpuscles.	[38]
7	Male Sprague-Dawley rats	4 mg/kg b.w./day	Dissolve in DMSO, diluted with saline	IV	24 hr/once	I/R and I/R+BPA groups had significant necrosis, nuclear pyknosis, and intercellular border loss.	[28]
8	Male Wistar rats	130 mg kg/b.w./day	Olive oil	Oral	4 weeks (28 days)	BPA-exposed rats exhibited dilated sinusoids, inflammatory cell infiltration, congestion, and necrosis.	[35]
9	Male C57BL/6 mice	1.0, 10, 50, and 250 *μ*g/kg b.w./day	PEG	Oral	35 days/daily/once	Liver appeared slight edema in the BPA group, but no other significant pathological changes.	[30]
10	Male Wistar rats	5 and 50 mg/kg b.w./day	Corn oil	Oral	5 weeks/daily/once	BPA-treated RIR group (BIR) showed dilated sinusoids, centrilobular congestion, and lymphocyte infiltration in the portal tract.	[31]

**Table 3:** Summary of selected studies on the mechanism of BPA-induced liver damage. Abbreviations: b.w.: body weight; GD: gestation day; IP: intraperiotoneal; NA: not applicable.

No.	Type of animal/cells	Dose/concentration	Mode of administration	Duration of treatment	Study type	Mechanisms	Reference
1	Specific pathogen-free C57BL/6 male mice	1.2 mg/kg b.w./day	IP	1, 6, and 24 hr	In vivo	Mitochondria dysfunction, increased inflammatory mediator production	[21]
2	Male Wistar albino rats	0.1, 1, 10, and 50 mg/kg b.w./day	Oral	4 weeks/daily/once	In vivo	Increased oxidative stress	[22]
3	Wistar albino rats	50 mg/kg b.w./day	Oral	8 weeks/daily/once	In vivo	Increased inflammatory mediator production	[23]
4	Virgin female (270–300 g) and male (350–400 g) genitor Wistar rats were	50 *μ*g/kg/b.w./day	Oral	GD 0 to the end of lactation at postnatal day 21	In vivo	Mitochondria dysfunction	[33]
5	Male Sprague-Dawley rats	2, 10, and 50 mg/kg b.w./day	IP	30 days; administered every 48 hr	In vivo	Increased oxidative stress	[42]
6	Male albino rats (Wistar strain)	150, 250, and 500 mg/kg b.w./day	Oral	14 days/daily/once	In vivo	Mitochondria dysfunction	[47]
7	Male CD-1 mice	50 *μ*g/kg/b.w./day	Oral	10 weeks/daily/once	In vivo	Mitochondria dysfunction, increased oxidative stress, increased inflammatory mediator production	[48]
8	NCTC Clone 1469	100 *μ*M	NA	48 hr	In vitro	Endoplasmic reticulum stress, increase oxidative stress	[2]
9	Female Swiss mice	70 *μ*g/kg b.w./day	Oral	3 months/daily	In vivo	Chronic endoplasmic reticulum stress	[56]

## Data Availability

The datasets supporting the conclusions of this study are included within the manuscript.
